# Understanding the aetiology and resolution of chronic otitis media from animal and human studies

**DOI:** 10.1242/dmm.029983

**Published:** 2017-11-01

**Authors:** Mahmood F. Bhutta, Ruth B. Thornton, Lea-Ann S. Kirkham, Joseph E. Kerschner, Michael T. Cheeseman

**Affiliations:** 1 Department of ENT, Brighton and Sussex University Hospitals NHS Trust, Brighton, BN2 5BE, England; 2 Division of Paediatrics, University of Western Australia, Subiaco, WA 6008, Australia; 3 Wesfarmers Centre of Vaccines and Infectious Diseases, Telethon Kids Institute, Subiaco, WA 6008, Australia; 4 Office of the Dean, Medical College of Wisconsin, Milwaukee, WI 53226, USA; 5 Division of Developmental Biology, Roslin Institute, University of Edinburgh, Midlothian, EH23 9RG, Scotland

**Keywords:** Chronic otitis media, Genetics, Animal models, Inflammation

## Abstract

Inflammation of the middle ear, known clinically as chronic otitis media, presents in different forms, such as chronic otitis media with effusion (COME; glue ear) and chronic suppurative otitis media (CSOM). These are highly prevalent diseases, especially in childhood, and lead to significant morbidity worldwide. However, much remains unclear about this disease, including its aetiology, initiation and perpetuation, and the relative roles of mucosal and leukocyte biology, pathogens, and Eustachian tube function. Chronic otitis media is commonly modelled in mice but most existing models only partially mimic human disease and many are syndromic. Nevertheless, these models have provided insights into potential disease mechanisms, and have implicated altered immune signalling, mucociliary function and Eustachian tube function as potential predisposing mechanisms. Clinical studies of chronic otitis media have yet to implicate a particular molecular pathway or mechanism, and current human genetic studies are underpowered. We also do not fully understand how existing interventions, such as tympanic membrane repair, work, nor how chronic otitis media spontaneously resolves. This Clinical Puzzle article describes our current knowledge of chronic otitis media and the existing research models for this condition. It also identifies unanswered questions about its pathogenesis and treatment, with the goal of advancing our understanding of this disease to aid the development of novel therapeutic interventions.

## Abstract (precis)

**Summary:** Chronic middle ear inflammation is a common disease. Animal models, and in particular mouse models, have been used to elucidate some potential mechanisms, including dysfunction in immune signalling, mucociliary function or Eustachian tube function.

## Introduction

**Fig. 1.**
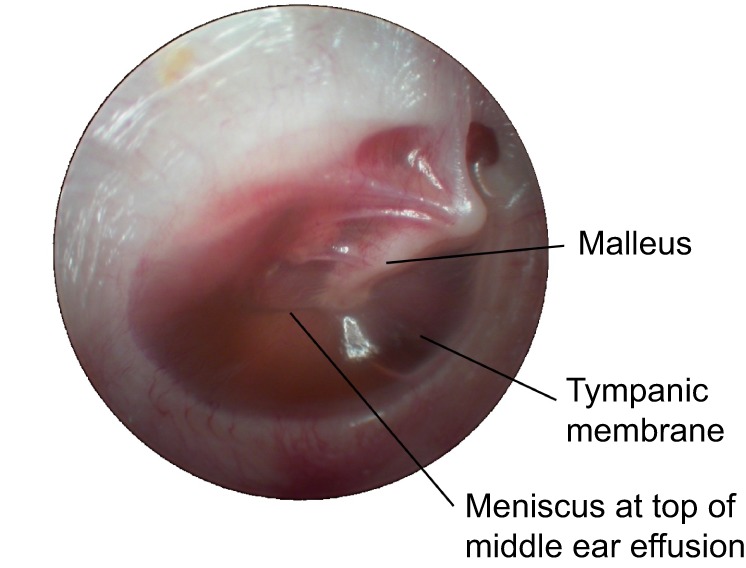
**Otoscopic view of the right ear of a 5-year-old child with COME (‘glue ear’).** An effusion is present behind (deep to) the tympanic membrane (ear drum), which appears as slight opacity. Image courtesy of Professor David Pothier, University of Toronto, Ontario, Canada.

Otitis media (OM; see Box 1 for a glossary of terms) describes an inflammatory disease of the middle ear that consists of a set of inter-related clinical phenotypes. Chronic otitis media with effusion (COME, or ‘glue ear’; Box 1) affects 5-6% of children in high-income countries in their second year of life (Bhutta, 2014), becomes less prevalent in older children (Suarez Nieto et al., 1983) and is rare in adults. COME is characterised by mucosal hyperplasia, including the proliferation of mucus-secreting goblet cells in the epithelial lining of the antero-inferior middle ear cleft. These changes lead to serous or mucoid middle ear effusion (Fig. 1, Box 2), which impairs the transmission of airborne sound. COME is the most common cause of hearing loss in childhood (Robb and Williamson, 2016).

**Box 1. Glossary of clinical terms**

**Acute otitis media (AOM):** an ear infection, usually accompanied by symptoms of fever and pain in the ear.

**Audiogram:** a test to measure hearing, with hearing thresholds measured in decibels.

**Bulla:** the middle ear cavity in animals.

**Chronic otitis media with effusion** **(COME):** otitis media with effusion present for at least 3 months.

**Chronic suppurative otitis media (CSOM):** chronic otitis media with a perforated tympanic membrane and intermittent or continuous otorrhoea. Duration of otorrhoea to define disease is debated: some suggest 2 weeks, others 6 weeks, others 3 months.

**Grommet:** a small, hollow (also called a ventilation) tube inserted into the tympanic membrane to treat symptomatic COME and resolve effusion.

**Myringoplasty:** an operation to repair the tympanic membrane.

**Myringotomy:** an operation to make an incision in the tympanic membrane.

**Otitis media (OM):** inflammation of the middle ear.

**Otitis media with effusion (OME):** serous or mucoid effusion in the middle ear, without signs or symptoms of infection. Usually the effusion is tenacious; hence, OME is colloquially called ‘glue ear’.

**Otorrhoea:** discharge from the ear.

**Otoscope:** an instrument to examine the ear.

**Recurrent AOM (rAOM):** usually defined as more than three episodes of AOM in 6 months, or more than four episodes in a year.

**Tympanic membrane:** the eardrum, a membrane between the middle and outer ear that vibrates in response to sound.

**Tympanometry:** a clinical test to evaluate the pressure of the middle ear or the presence of fluid by observing the mobility of the eardrum in response to induced variations in the air pressure in the ear canal.

**Box 2. Case study**

A 5-year-old boy is taken to the doctor by his parents because both they and his schoolteachers have noticed that for several months his hearing seems poor. He is also behind his peer group in his spoken and written language, and there are concerns about poor behaviour. There is no preceding history of ear infections and the boy is otherwise well. The boy's father also had hearing problems in childhood. Examination with an otoscope reveals evidence of effusion behind the tympanic membrane (see Fig. 1), giving a diagnosis of otitis media with effusion (OME; or ‘glue ear’). This is confirmed by tympanometry. An audiogram reveals a 40-decibel hearing loss. After a discussion of the options, the parents decide to proceed with bilateral grommet insertion under general anaesthesia, which normalises hearing and leads to improvements in language development and behaviour.

After a year, the grommets fall out, but the left tympanic membrane has a residual perforation. Subsequently, the child suffers recurrent left-sided mucopurulent otorrhoea, which occurs every few months. The child is repeatedly treated with topical antibiotic drops but is unable to continue with his swimming lessons. At the age of 8 years, he undergoes a left myringoplasty, which successfully repairs the tympanic membrane. The child has no further problems. (See Box 1 for a glossary of clinical terms.)

**Fig. 2.**
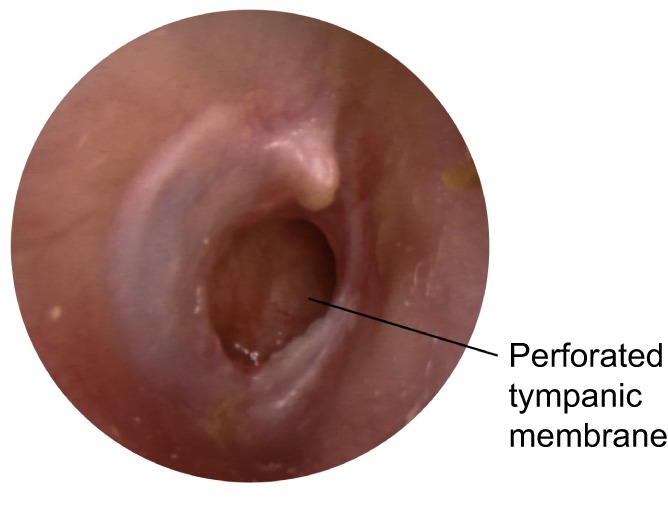
**Otoscopic view of the right ear of a child with CSOM.** The image shows a perforation of the posterior tympanic membrane. This patient complained of intermittent ear discharge (otorrhoea), although at the time of this image no discharge is evident. Image courtesy of Professor David Pothier, University of Toronto, Ontario, Canada.

Chronic suppurative otitis media (CSOM; Box 1) is characterised by a persistent perforation of the tympanic membrane (Box 1, Fig. 2) with intermittent or constant discharge of pus through this perforation (a condition known as otorrhoea; Box 1 and see the accompanying case study in Box 2). CSOM is rare (<1%) in high-income countries but relatively common (>2%) in many low- and middle-income countries, and is highly prevalent (>4%) in some indigenous groups, such as in Australian Aboriginal, Pacific Islander, Native American and Inuit populations (Bhutta, 2015). CSOM is estimated to affect 65-million–330-million people worldwide (WHO, 2004). Epidemiological studies indicate that the highest incidence of CSOM occurs in childhood (Monasta et al., 2012), but others have suggested that prevalence continues to rise into adulthood (Shaheen et al., 2012; Chung et al., 2016).

**Fig. 3.**
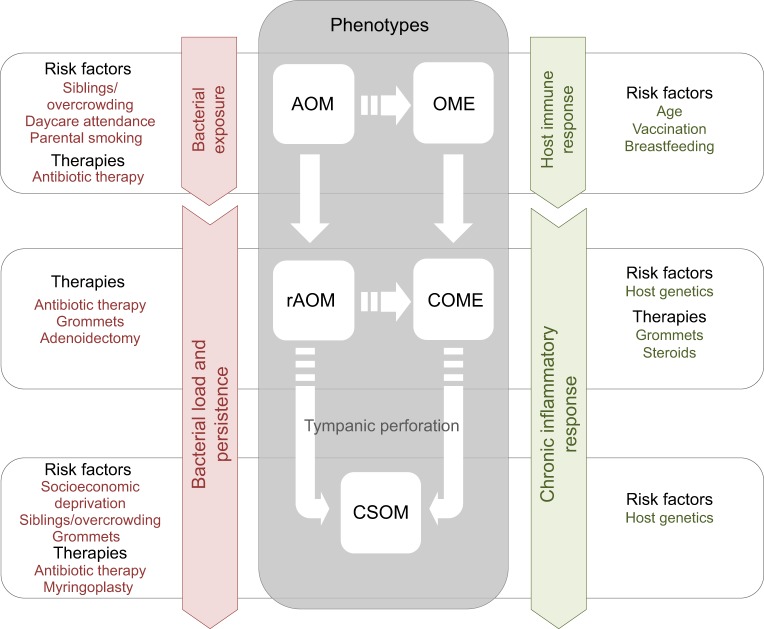
**Inter-related phenotypes of mucosal OM, together with their known or presumed risk factors.** In this schematic, white broken arrows denote postulated links between OM-related conditions and unbroken arrows represent known links. Risk factors and therapies associated with pathogen exposure (left) and with host characteristics and inflammatory response (right) for acute and chronic forms of OM are shown. There are two forms of chronic mucosal OM: COME (chronic OM with effusion) and CSOM (chronic suppurative OM). Note that OME and COME can occur without antecedent AOM. The causes of CSOM are not well understood. AOM, acute otitis media; rAOM, recurrent AOM.

The risk of developing either COME or CSOM involves a complex interplay between host immunity and microbial pathogenicity, which, in turn, is affected by host and microbe genetics, as well as by environmental factors (particularly those that affect risk of exposure to bacteria) and by therapeutic interventions (Fig. 3). Most cases of COME are preceded by a bacterial or viral infection of the middle ear, which causes acute otitis media (AOM; Boxes 1, 2). After an episode of AOM, the middle ear effusion becomes non-purulent [otitis media with effusion (OME); Box 1] and then usually resolves within days; however, in an estimated 8% of affected children it persists for more than 3 months and becomes chronic (COME) (Bhutta, 2014). What distinguishes the minority of children who develop chronic inflammation from those that do not is a key research question. Recurrent AOM (rAOM; Box 1) is also a risk factor for developing COME (Alho et al., 1995), but most children with COME do not suffer from rAOM.

Relatively little is known about the aetiology of CSOM, but it is thought to occur as a consequence of recurrent or persistent middle ear inflammation, which leads to a non-healing perforation of the tympanic membrane. Many affected individuals present without a history of preceding symptoms, but there is evidence that early or recurrent AOM (Fliss et al., 1991; Lasisi et al., 2007; van der Veen et al., 2006), or COME (Youngs, 1998), increases an individual's risk of developing CSOM. Chronic otorrhoea can also develop in children after the insertion of grommets (Box 1).

In this Clinical Puzzle, we discuss our current knowledge of chronic OM and the existing research models that have been generated to investigate the factors that contribute to this disease. We also identify unanswered questions about the pathogenesis and treatment of these chronic ear conditions, and highlight the need for us to advance our understanding of the aetiology and biology of this disease in order to develop novel therapeutic interventions for this prevalent and chronic condition.

## Chronic otitis media can resolve

What is unusual about both COME and CSOM is that these conditions can resolve spontaneously. Many other chronic inflammatory disorders, such as rheumatoid arthritis or multiple sclerosis, demonstrate a clinical course of persistent or recurrent inflammation, and rarely result in permanent resolution (Nathan and Ding, 2010).

For patients with COME, effusion can be resolved through the insertion of grommets (ventilation tubes), although the mechanism underlying this effect is unknown. Around one fifth of children treated with grommets in infancy will have a recurrence of effusion once the grommets extrude from the tympanic membrane, yet, by the age of 6 years, hearing will have normalised in almost all affected children, irrespective of treatment (Johnston et al., 2004; Khodaverdi et al., 2013).

Resolution can also occur in CSOM, and healing of the tympanic membrane has been noted in some patients after treatment with topical antibiotics (Gupta et al., 2014; Smith et al., 1996). However, spontaneous resolution occurs less commonly than in cases of COME. In a study of 549 children in Greenland, 9% were found to have CSOM and, when a subset of this cohort was followed up after 15 years, the tympanic membrane had healed in only one third (Jensen et al., 2012).

## Existing model systems for chronic otitis media

**Fig. 4.**
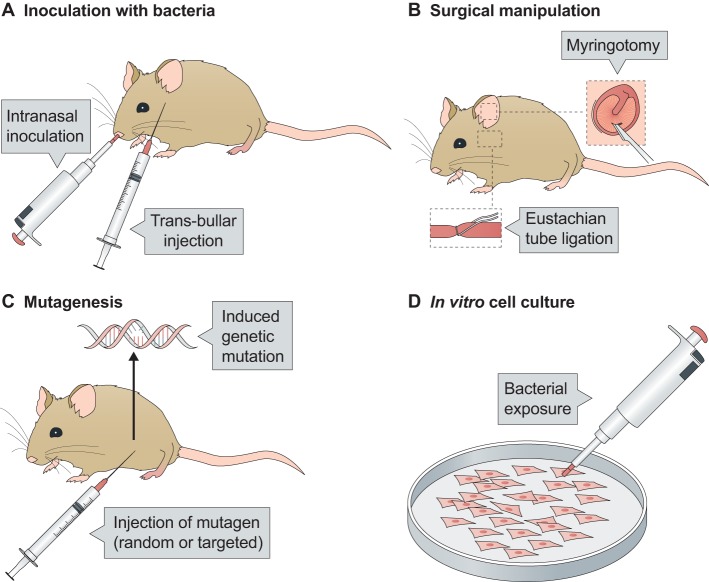
**Common methods to induce acute or chronic OM in mouse models.** Methods shown here are for mouse models (methods to induce acute or chronic otitis media are determined by the model species and the hypothesis under test, and can be combined; for example, inoculating a genetic mutant with bacteria). (A) Inoculation with bacteria. A mouse is inoculated with bacteria, intranasally or by injection directly into the middle ear (bulla). Bacteria commonly used include pneumococcus or NTHi. (B) Surgical methods. The top inset shows surgically induced tympanic membrane perforation, and the lower inset shows surgical ligation of the Eustachian tube. (C) Genetic manipulation. This can be performed via chemical mutagenesis [typically with injection of the chemical mutagen N-ethyl-N-nitrosourea (ENU), as shown] or by targeted genetic mutation. (D) *In vitro* cell culture, for example of immortalised middle ear epithelial cells or mucosal explants. Following exposure to bacteria, these cell-culture models enable host-pathogen interactions at the epithelial surface to be assessed.

The mouse has become the preferred animal model for OM research owing to the availability of suitable reagents, low husbandry costs, genetic tractability, a well-characterised immune response and well-defined microbiological status (Fig. 4) (Bhutta, 2012). The long-tailed chinchilla has also been used for OM research because its large middle ear and Eustachian tube more closely resemble the anatomy of humans, and make it easier to recover effusion for microbiological or immunological analysis (Doyle, 1985; Jurcisek et al., 2003). For these reasons, the chinchilla has proven to be especially useful in vaccine studies and, because the chinchilla is outbred, it also better models the heterogeneity of immunological response compared to inbred animals (Green et al., 1994; Novotny et al., 2013). Other rodents used to study OM include rats, guinea pigs and gerbils (Bhutta, 2012; Sabirov and Metzger, 2008). Non-rodent animal models are rarely used to study this condition.

*In vitro* cell-culture models to study OM have also been developed using immortalised human middle ear epithelial cells (Chun et al., 2002), rat mucosal explants (Hill et al., 1992) and murine primary middle ear epithelial cells (Mulay et al., 2016; Tsuchiya et al., 2005) (Fig. 4D). Although these cell-culture models enable host-pathogen interactions to be assessed at the epithelial surface (Samuel et al., 2008; Palacios et al., 2004; Val et al., 2016a), they cannot fully recapitulate the complexity of host-pathogen interaction *in vivo*, which is needed to understand the pathophysiology of chronic OM.

There are currently no experimental models that fully replicate the development or progression of chronic OM in humans. AOM is induced in animal models either by injection of bacteria directly into the bulla (Box 1; Oishi et al., 2013) or by intranasal inoculation coupled with viral co-infection (Langereis et al., 2012) or nasal pressurisation to facilitate ascension of bacteria from the nose to the middle ear (Chaney et al., 2011; Fig. 4A). The injection of less-virulent bacteria or of a reduced bacterial load leads to histological changes that resemble OME rather than AOM (Hermansson et al., 1988). In such models, inflammation is short-lived, the infection resolves within days and transition from AOM to chronic OM is not observed. Chronic OM (without preceding AOM) can be induced in mice or rats either through surgical obstruction of the Eustachian tube (Varsak and Santa Maria, 2016; Santa Maria et al., 2015; Hirano et al., 2016) or through genetic mutation (Zheng et al., 2006) (Fig. 4B,C).

Current genetic mutant mouse models of chronic OM are listed in Table S1 and reveal that a wide range of biological mechanisms can result in chronic OM in mice. OM penetrance ranges from ∼30% in the *BpifA1*-null mouse mutant (*BpifA1* encodes the innate immune response protein BPI fold-containing family A member 1) (Bartlett et al., 2015) to ∼80% in a mouse carrying a point mutation at the immunomodulatory *Mecom* (MDS1 and EVI1 complex) locus (Parkinson et al., 2006). OM mouse models recapitulate many of the features of human chronic OM. The effusion that accumulates in the middle ear (bulla) varies from serous in mice that carry a point mutation at the protein regulatory locus *Fbxo11* (F-box protein 11) (Hardisty-Hughes et al., 2006) to purulent in *Mecom* mutant mice, and features variable proportions of polymorphonuclear cells (including foamy large macrophages), lymphocytes, plasma cells and apoptotic or necrotic cells. In these models, the inflamed bulla mucosa is thickened, oedematous and often bears polyps, with capillary and lymphatic proliferation, and often a loss of ciliated cells and increased goblet cell number (Parkinson et al., 2006; Hardisty et al., 2003). Mucosal fibrosis has also been noted in *Mecom* mutants, *BpifA1* mutants and in *Oxgr1*-null mice (*Oxgr1* encodes the G-protein-coupled receptor oxoglutarate receptor 1), and in mice carrying a point mutation in the pattern-recognition receptor *Tlr4* (Toll-like receptor 4) (Kerschner et al., 2013; MacArthur et al., 2006). Cholesterol granulomas are seen in mice with a semi-dominant point mutation in the gene encoding ribosomal protein L38 (*Rpl38*) and in mice with a semi-dominant mutation in the gene encoding the nuclear scaffold lamin A/C proteins (*Lmna*) (Noben-Trauth and Latoche, 2011; Zhang et al., 2012). Foreign-body granulomas have also been found in mice null for ectodysplasin A (*Eda*) and its receptor (*Edar*), which are involved in ectodermal morphogenesis (Azar et al., 2016).

It is important to note that the pathology of mouse models also differs from certain aspects of human COME and CSOM. No mouse mutant develops the tenacious fluid that typifies the effusion of the human mucoid form of COME, although there is evidence of a modest increase in mucus production. Mucin genes are upregulated in the mucosa of the *Oxgr1* mouse mutant, and there is goblet cell hyperplasia in *Lmna*, *Eda* and *Edar* mutants. Goblet cell hyperplasia is also seen with OM in mice carrying a null mutation in the following genes: the chromatin-remodelling gene *Chd7* (chromodomain-helicase-DNA-binding protein 7), the transcriptional co-activator *Eya4* (EYA transcriptional coactivator and phosphatase 4), the immunomodulatory gene *Tgif* (TGFB induced factor homeobox 1) and the structural protein *Sh3pxd2b* (SH3 and PX domains 2B) (Tian et al., 2012; Depreux et al., 2008; Tateossian et al., 2013; Yang et al., 2011). Goblet cell hyperplasia also occurs as a result of chromosomal microdeletion in the *Df1* mouse model of the human 22q11 deletion syndrome (Fuchs et al., 2013). Importantly, COME in children may resolve either spontaneously or after successful grommet treatment, whereas spontaneous remission of chronic OM is not documented in animal models.

The hallmark features of human CSOM, namely tympanic membrane perforation and purulent otorrhoea, are uncommon sequelae in genetic mouse models. In the *Mecom* mutant, otorrhoea occurs in conventionally housed low-health-status mice over 6 months of age but not in high-health-status specific-pathogen-free (SPF) conditions (Parkinson et al., 2006). Many other mouse OM models have not been assessed at this age and so it is possible that they could also develop otorrhoea if allowed to age. The findings in the *Mecom* mouse support the argument that laboratory mice should experience more normal environmental exposure to natural pathogens in order to better model human microbial exposure (Beura et al., 2016).

Several authors have attempted to induce CSOM in rodents through surgical means. In wild-type mice, surgical tympanic membrane perforation followed by the introduction of infection does not lead to chronic otorrhoea, and the tympanic membrane usually heals (Wang et al., 2014). Tympanic perforation in mutant *Mecom* mice also heals within 5 days, despite the presence of a pre-existing chronic purulent effusion (Bhutta et al., 2014). CSOM can be reliably induced in mice and rats by a combination of tympanic membrane perforation, blockade of the Eustachian tube, prevention of tympanic membrane healing (through grommet insertion or through the application of the matrix metalloprotease inhibitor KB-R7785) and by infection with *Pseudomonas aeruginosa* or *Streptococcus pneumoniae* (Santa Maria et al., 2015; Silva et al., 2012). It is still uncertain whether these are good models of CSOM because, in human disease, the Eustachian tube is usually normal or only partially obstructed (Bhat et al., 2009). Further studies of disease pathogenesis with these CSOM models are warranted.

Chronic OM is a feature of human syndromic conditions, such as Down syndrome, hypohidrotic ectodermal dysplasia (HED), primary ciliary dyskinesia (PCD), mucopolysaccharidosis and 22q11.2 deletion syndrome, and is also observed in the mouse strains that bear the comparable genetic lesions (Table S1). Other current mouse models of chronic OM have syndromic features, and so the pathology and mechanisms described in these models must be translated cautiously to non-syndromic disease in humans. In addition, some syndromic mouse models, such as perinatal lethal neurofibromatosis type 2, have OM (Giovannini et al., 2000), but this is not a feature of the equivalent human condition.

## The role of pathogens in chronic otitis media

**Fig. 5.**
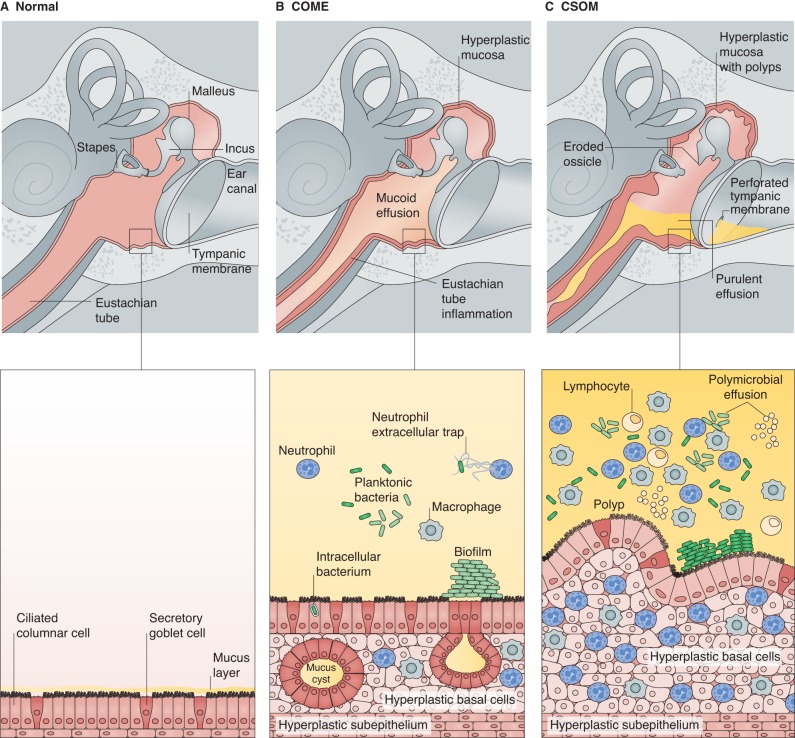
**Gross and microscopic pathology in different subtypes of chronic OM.** (A) The structures of a child's middle ear, including the ossicles (malleus, incus and stapes), tympanic membrane and Eustachian tube. The normal ciliated and non-ciliated epithelial lining of the middle ear, overlaid by a thin layer of surface mucus, is shown in a magnified view (lower panel). (B) The middle ear of a child with COME. The pale yellow shading depicts mucoid effusion. Lower panel: the inflamed lining of the middle ear features mucosal hyperplasia with secretory goblet cell proliferation. Bacteria can exist in the effusion in a planktonic state, in biofilms or intracellularly, and neutrophils and macrophages are present. (C) The middle ear of a child with CSOM. Yellow shading depicts purulent effusion, and the tympanic membrane is perforated. Lower panel: the inflamed lining of the middle ear features mucosal hyperplasia, which can be profuse and form polyps. Bacteria are present in a variety of forms and many different bacterial species can be found. Neutrophils, macrophages and lymphocytes are present in abundance.

COME is often preceded by AOM, and so it is likely that bacterial and/or viral pathogens initiate inflammation in such cases (Fig. 5). It is generally accepted that infection with an upper respiratory virus often precedes bacterial AOM. The main bacteria that cause AOM are *S. pneumoniae* (pneumococcus), non-typeable *Haemophilus influenzae* (NTHi) and *Moraxella cattarrhalis* (Ngo et al., 2016)*.* These bacteria are also found in the middle ear effusion of children with chronic OM, but microbiome studies reveal that a multitude of other bacterial species are also present, in both COME (Chan et al., 2016; Jervis-Bardy et al., 2015) and CSOM (Neeff et al., 2016).

The role of bacteria in contributing to the persistence of inflammation in COME is not clear. There is some evidence that children who have recurrent episodes of AOM are more likely to have middle ear effusion (Alho et al., 1995) but it is uncertain whether this represents repeated episodes of acute resolving effusion or continuous non-resolving effusion. Different studies using culture or molecular identification have detected bacterial DNA in 14-73% of effusions from children with COME (this wide range may reflect differing sensitivity of detection methods), and NTHi is more likely to be present than is pneumococcus (Ngo et al., 2016). Live bacteria have been found in a biofilm matrix on mucosal surfaces and/or in middle ear effusion (Thornton et al., 2013; Hall-Stoodley et al., 2006), intracellularly within mucosal cells and planktonically within the effusion (Thornton et al., 2011) (Fig. 5B,C). There is evidence that the effusion in COME is more likely to resolve in children who are given antibiotics (Venekamp et al., 2016), although that effect is relatively small.

In CSOM, bacteria play a more definite role in disease perpetuation. The prevalence of CSOM correlates with socioeconomic deprivation and malnutrition, both between and within countries (Bhutta, 2014; Lasisi et al., 2007; Chadha et al., 2006; Shaheen et al., 2012; Ologe and Nwawolo, 2003). These factors likely have an impact on immune responses, on the risk of pathogen exposure and on pathogen load. Microbiological culture of middle ear effusion from children with CSOM yields a mix of aerobic and anaerobic bacteria (Verhoeff et al., 2006), usually with a predominance of *Staphylococcus aureus* and *P. aeruginosa* (Mittal et al., 2015). Bacteria in CSOM also exist in a biofilm (Lee et al., 2009; Saunders et al., 2011; Gu et al., 2014; Homøe et al., 2009). Topical or oral antibiotics may stop otorrhoea in patients with CSOM, but treatment will frequently fail and it is also not known how often antibiotic treatment enables the long-term resolution of disease, including healing of the tympanic membrane (Macfadyen et al., 2005, 2006).

All chronic OM mouse mutants develop disease spontaneously without the need for bacterial challenge. Wild-type SPF mice have a diverse nasal microbiome (Krone et al., 2014) and nasal commensals are a potential source for bulla infection. Nasal commensals have been cultured from the bullae of various mouse mutants that carry null mutations, including in genes involved in ectodermal morphogenesis (*Eda*, *Edar* and *Mcph*, which encodes microcephalin) (Azar et al., 2016; Chen et al., 2013), in chromatin remodelling (*Chd7*) (Tian et al., 2012), in transcription (*Eya4*, *Df1* and *Isl1*, which encodes ISL LIM homeobox 1) (Depreux et al., 2008; Hilton et al., 2011; Fuchs et al., 2013), in protein synthesis (*Rpl38*) (Noben-Trauth and Latoche, 2011) and in immune signalling (*Mecom* and *Nfkbia*, which encodes NFKB inhibitor alpha) (Schmidt-Ullrich et al., 2001; Parkinson et al., 2006). However, not all bulla fluids are culture positive (Table S1). The human otopathogens NTHi, pneumococcus and *Moraxella catarrhalis* have not been detected by PCR in *Chd7* or *Oxgr1* mouse mutants, but *M. catarrhalis* was detected in the *Spag6* (sperm-associated antigen 6)-null mutant, which has defects thought to result from disruption of the ciliary cytoskeleton (Li et al., 2014). Anaerobic culture and microbiome studies have yet to be performed in these OM mutants. Antimicrobial (azithromycin) treatment does not suppress OM in *Eya4* mutants (Depreux et al., 2008). In *Mecom* mutants, OM initiates later, but occurs with the same frequency under germ-free and SPF conditions, suggesting that respiratory irritants, such as ammonia and dust from the cage environment, can act as inflammatory stimuli to initiate OM. Intranasal challenge of *Mecom* mutants with NTHi results in high rates of bulla infection lasting up to 56 days, and shows the potential of this model for treatment and prevention studies for chronic OM. There is no evidence of NTHi intraepithelial infection or biofilm formation in *Mecom* mice (Hood et al., 2016).

## The role of host factors in chronic otitis media

The relative importance of host factors in the initiation and perpetuation of chronic OM can be gauged from estimates of the heritability of disease. Twin studies in young children with COME have suggested that heritability of the duration of middle ear effusion is high at 0.73 (Casselbrant et al., 1999). Studies of the Inuit population in Greenland report that parental history is an important predictor of CSOM in children, independent of socioeconomic status (Jensen et al., 2011; Koch et al., 2011).

Identifying genetic loci that are associated with chronic OM is one way in which to elucidate potential disease mechanisms. A number of human genetic-association studies for OM have been reported (reviewed in Rye et al., 2012a), but most early studies comprised small cohorts (and so were underpowered or at high risk of false discovery) and, in many, the condition was poorly defined (Bhutta, 2013). More recent studies have featured larger cohorts and better phenotyping (Table 1) but, nevertheless, they remain too small to discover loci that are associated with modest relative risk. The only OM association to have been replicated is at the *FBXO11* locus. *FBXO11* was initially associated with OM in an Australian cohort (Rye et al., 2011), with nominal evidence of association, and then replicated in a UK cohort (Bhutta et al., 2017) and a US cohort (Segade et al., 2006), albeit at different polymorphisms at *FBXO11*. Data from the *Fbxo11* mouse model (see below) suggest that a mutation in *Fbxo11* perturbs transforming growth factor (TGF)-β signalling in the middle ear.

**Table 1.**
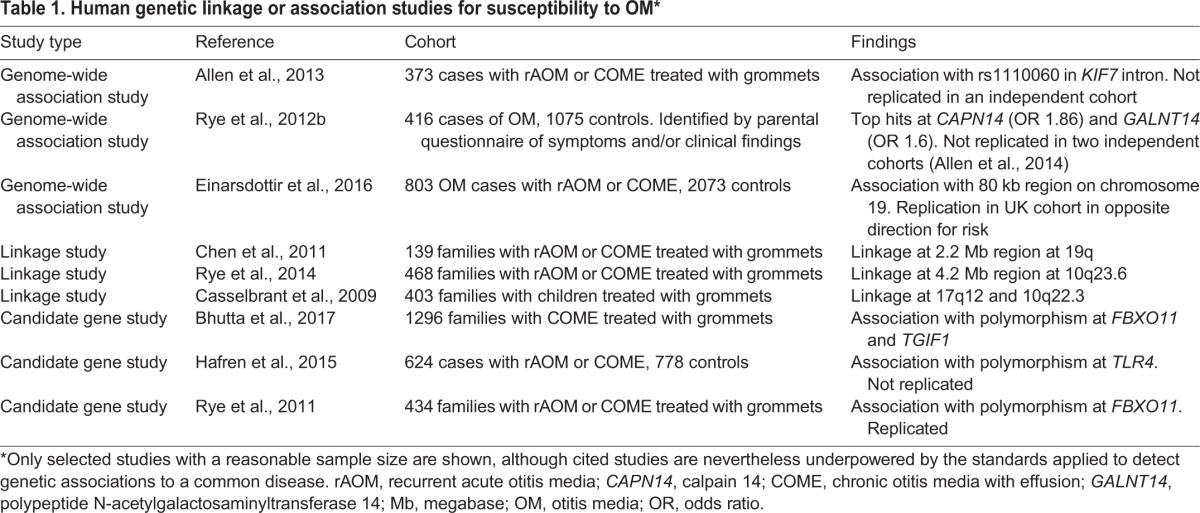
Human genetic linkage or association studies for susceptibility to OM*

The factors that contribute to host susceptibility to chronic OM have also not been elucidated. The onset of AOM is presumed to involve the detection of (bacterial) antigens by innate immune receptors (such as the Toll-like receptors) and by other pattern-recognition molecules on cells within the middle ear mucosa, which will recruit neutrophils and lymphocytes to the middle ear. However, we do not know whether it is signalling and regulation by the mucosa, leukocytes, or both, that perpetuates inflammation in the chronic condition, and whether the inflammatory mechanisms involved are organ-specific or mirror mechanisms of chronic inflammation found in other tissues (Buckley et al., 2013; Val et al., 2016b).

Much of the older literature suggests that poor aeration of the middle ear due to ventilatory dysfunction of the Eustachian tube is a key factor in acute and chronic OM (Bluestone, 2005), yet there are no validated tests of Eustachian tube function to support this notion (Smith and Tysome, 2015). Moreover, anatomical modelling suggests that a narrowing of the Eustachian tube is unlikely to significantly impede gas exchange (Sadé et al., 2004). Additionally, no consistent or significant differences in Eustachian tube parameters have been demonstrated in individuals affected by OM (Sadé and Luntz, 1989; Sadé et al., 1986). However, in *Df1* and *Tbx1* (T-box 1)-null mouse mutants (which model human 22q11 deletion syndrome), developmental hypoplasia of the levator veli palatini muscle, which is involved in opening and closing the anterior pharyngeal portion of the Eustachian tube, impairs the experimental transit of dye from the bulla (Fuchs et al., 2013; Liao et al., 2004). In *Fbxo11* (Hardisty et al., 2003) and *Eya4* (Depreux et al., 2008) mutants, the Eustachian tube can be malpositioned, narrowed or misshapen and, in *Eya4* mutants, its opening can be blocked by inflammatory polyps. An adrenergic signalling defect in *Dbh* (dopamine beta-hydroxylase) mutants might also impair tubal function (Maison et al., 2010). In addition, mouse mutants with craniofacial abnormalities, such as domed heads and alterations in the cranial base, can have Eustachian tube anomalies; for example, the angle at which the tubes join the nasopharynx is more acute in *Sh3pxd2b*, *Lmna* and *Chd7* mutants (Zhang et al., 2012; Yang et al., 2011; Tian et al., 2012). In *Rpl38* and *Edar* mutants, the Eustachian tube undergoes pathological dilation through loss of adjacent submucosal glands (Azar et al., 2016; Noben-Trauth and Latoche, 2011), and, in *Eda* and *Edar* mutants, the gating function of the Eustachian tube is reduced, permitting larger foreign-body particles to enter the bulla (Azar et al., 2016). There are anatomical defects in the middle ear of other mouse mutants that could also be relevant; for example, defective postnatal bulla cavitation in the *Eya4* null mutant (Depreux et al., 2008), hypoplastic but proportionate skulls in the microcephaly-associated *Mcph1*-null mutant (Chen et al., 2013), shortened and distorted nasal bones with small bullae in compound mutants of the transcription factors *Ets1* (ETS proto-oncogene 1, transcription factor) and *Fli1* (Fli-1 proto-oncogene, ETS transcription factor) (Carpinelli et al., 2015), and perturbation of epithelial growth in the *Fbxo11* and *Tgif* mutants (Hardisty et al., 2003; Tateossian et al., 2013).

The importance of mucociliary defects in the perpetuation of middle ear inflammation is not known. In primary cilia dyskinesia (PCD) syndrome, patients have an increased incidence of middle ear effusion, and mouse mutants with null mutations in the following ciliary structural proteins have OM and rhinitis due to impaired mucociliary clearance: *Dnah5* (dynein axonemal heavy chain 5), *Cby1* (chibby family member 1, beta catenin antagonist), *Spag6*, *Dnah11* (dynein axonemal heavy chain 11), *Odf2* (outer dense fiber of sperm tails 2), *Ttll1* (tubulin tyrosine ligase-like 1), *Ulk4* (unc-51-like kinase 4), *Kif27* (kinesin family member 27), *Dpcd* (deleted in primary ciliary dyskinesia homolog) and *Stk36* (serine/threonine kinase 36) (Ibanez-Tallon et al., 2002; Voronina et al., 2009; Li et al., 2014; Lucas et al., 2012; Kunimoto et al., 2012; Vogel et al., 2010, 2012). *Mcph1* mutant mice are also suspected to have cilia defects, as well as the null mutant at *Porcn* (porcupine O-acyltransferase), a gene involved in the processing of proteins by the endoplasmic reticulum (Chen et al., 2013; Biechele et al., 2013). In *Lmna*, *Chd7*, *Eya4* and *Phex* (phosphate regulating endopeptidase homolog, X-linked) mutants, inflammation causes the loss of ciliated cells in the bulla epithelium, whereas perturbation of phosphorylation in the fibroblast growth factor (FGF)23/prostaglandin (PG)E2 pathways in the *Phex* hypomorph might also reduce the aqueous periciliary layer and impair the clearance of overlying mucus (Han et al., 2012; Zhang et al., 2012; Tian et al., 2012; Depreux et al., 2008).

## Mechanisms leading to chronic inflammation and resolution

What enables chronic OM to resolve is unclear, but this seems an important avenue of research. This is because, by better understanding the clearance mechanisms that lead to resolution, we might be able to clinically manipulate the disease to hasten resolution, and thereby reduce disability.

At a molecular level, the resolution of inflammation in tissues other than the middle ear is complex, involving the depletion of chemokines, the downregulation of pro-inflammatory cytokines, the upregulation of pro-resolution mediators, neutrophil apoptosis and the alternative activation of macrophages (Sugimoto et al., 2016). It seems likely that similar mechanisms will operate in mucosal cells and in leukocytes to resolve inflammation in the chronically inflamed middle ear, but which of these mechanisms is important, and how they are regulated, is not known.

Existing clinical treatments for chronic OM can be highly effective, but investigation into their mechanisms of action has been limited. In COME, symptoms result from effusion, and the insertion of grommets is effective in resolving that effusion (Browning et al., 2010). Grommets are often presumed to have a rheological effect but there is no evidence that grommets affect the ventilatory function of the Eustachian tube in the short (van der Avoort et al., 2009), medium (Takahashi et al., 1990; van Heerbeek et al., 2001; Straetemans et al., 2005), or long (Cayé-Thomasen et al., 2008) term. Myringotomy (Box 1) in a mouse model reduced tissue hypoxia and inflammatory effusion, suggesting that oxygen tension is important for middle ear homeostasis, and this might be an alternative explanation for the efficacy of grommets (Bhutta et al., 2014). Once grommets extrude, middle ear effusion can recur, leading to a repeat operation in one quarter of treated children (Browning et al., 2010). This suggests that grommets have little long-term effect on the goblet cell hyperplasia that generates middle ear effusion.

In CSOM, symptoms result from perforation of the tympanic membrane, which is associated with ongoing intermittent or chronic inflammatory and infected otorrhoea. Mechanisms underlying the healing or non-healing of the tympanic membrane are not well understood (Jung et al., 2013) but, where the tympanic membrane does not heal spontaneously or following medical treatment with antibiotics, surgical repair is often successful. Nevertheless, one in six attempts at surgical repair will fail, which may be due to a number of factors; however, evidence of continuing chronic inflammation in the contralateral ear (in the form of COME) has been shown to be predictive of failure (Hardman et al., 2015).

Molecular targeting may offer more reliable clinical resolution of chronic OM in the future. This notion is still some distance from the bedside, but mouse models have suggested pathways that could be targeted. For example, genome-wide transcriptional analysis of acute OM, induced in mice through transbullar injection, reveals an early response at 6 h that is dominated by immune and defence proteins, and a later response at 24-48 h that is dominated by immunoregulatory proteins (Hernandez et al., 2015). However, inflammation resolves within 5-7 days of inoculation, and so this model provides little insight into the molecular signatures that underlie the transition to chronic disease. In mouse mutants, deficits in innate immunity are known to contribute to persistent inflammation. In the *Tlr4* mouse mutant, this occurs via altered responses to Gram-negative bacteria and, unusually for mouse mutants, the inflammatory changes extend into the inner ear (MacArthur et al., 2006). Other examples include the *BpifA1* mutant, in which the loss of the antimicrobial/surfactant protein product SPLUNC1 impairs auditory tube function (Bartlett et al., 2015), and the hypohidrotic ectodermal dysplasia (HED) mutants *IkB^αΔN^*, *Edar^dlJ/dlJ^*, *Eda^Ta/Y^* and *Eda^Ta/Ta^*, in which the nasal and nasopharyngeal glands and their products are absent (Schmidt-Ullrich et al., 2001; Azar et al., 2016). Impaired bulla mucosa secretion and response to Gram-negative bacteria is predicted in the *Isl1* mutant owing to the known interaction of this gene with innate immune signalling pathways (Hilton et al., 2011). Impaired adrenergic signalling in *Dbh* mutants might also affect the systemic and mucosal adaptive immune system (Maison et al., 2010).

Other models have suggested that perturbation of the TGF-β, NF-κB or HIF (hypoxia-inducible factor) signalling pathways can drive chronic OM. In *Fbxo11^Jf/+^* and *Tgif1^−/−^* mutants, TGF-β signalling is disrupted (Tateossian et al., 2015, 2013). TGF-β regulates the differentiation, proliferation and activation of several immune cells and, in sites other than the middle ear, persistent TGF-β activation has been found to promote the transition from acute to chronic inflammation, including fibrosis (Yoshimura et al., 2010). TGF-β levels also correlate with duration of effusion in children with COME (Zhao et al., 2009). Both HIF and NF-κB signalling are activated in response to cellular stress, which is induced by pathogens but also by chemical or physical damage (via NF-κB signalling) or cellular hypoxia (via HIF signalling). There is considerable crosstalk between these pathways (D'Ignazio et al., 2016) and they induce inflammation as part of a strategy to promote cellular survival, but persistent activation can lead to non-resolving inflammation. MECOM acts as an inducible negative regulator of NF-κB, and loss of *Mecom* function in mice results in an elevated response to inflammatory stimuli, including to challenge with NTHi (Xu et al., 2012). In *Mecom*, *Fbxo11* and *Tgif* mutant mice, leukocytes in the bulla fluid respond to inflammatory hypoxia by upregulating VEGF (vascular endothelial growth factor), a downstream effector in the HIF pathway. In *Mecom* mutants, tissue hypoxia extends to the mucosa, suggesting that hypoxia might be a common finding in the chronically inflamed middle ear (Cheeseman et al., 2011). HIF signalling was also upregulated in response to Eustachian tube blockage in a rat model of OM (Huang et al., 2012). VEGF signalling has been demonstrated in effusions of children with COME, and NF-κB in the mucosa of patients with CSOM (Sekiyama et al., 2011; Jesic et al., 2014).

Systemic administration of VEGF-receptor antagonists has been shown to ameliorate the progression of hearing loss in young *Mecom* mice before chronic OM is established (Cheeseman et al., 2011). Molecules to target NF-κB and TGF-β pathways have also been developed (Gilmore and Garbati, 2011; Fabregat et al., 2014) but have yet to be trialled for chronic OM.

## Conclusions and future outlook

Despite the considerable prevalence of chronic OM worldwide, especially in childhood, many unanswered questions remain about its aetiology and about how to treat this disease (as summarised in Box 3). In terms of epidemiology, existing studies provide some understanding of the microbiological and host factors that predispose children to COME, but we do not understand what initiates the disease in those without a history of AOM, nor whether CSOM is a more severe variant of COME.

**Box 3. Outstanding clinical and basic research questions**

- What leads to CSOM? Longitudinal epidemiological studies of OM are needed to address this question.
- What are the relative roles of mucosal biology, leukocyte biology, pathogens and Eustachian tube function in the perpetuation and resolution of COME and CSOM?
- How do we generate larger and well-phenotyped cohorts for genetic studies into human chronic OM?
- How do we create new and better animal models of chronic OM, and better evaluate their phenotypic relevance to human disease?
- How should we investigate the mechanisms that underlie current therapeutic interventions, including the long-term effects of antibiotic therapy for CSOM, and the immunobiological effects of perforation or repair of the tympanic membrane?

In terms of elucidating the pathobiology of chronic OM, we still need to understand the relative roles of mucosal versus leukocyte biology in the initiation and perpetuation of middle ear disease, and the role of pathogens and their interaction with host tissues. We do not know whether ventilatory dysfunction in the Eustachian tube is as important a mechanism in pathogenesis as was historically proposed (Bluestone, 2005). This question has become more pertinent with the recent development of balloons, which are used to surgically dilate the Eustachian tube with the aim of permanently improving ventilation of the middle ear in individuals with chronic middle ear disease (Miller and Elhassan, 2013; Norman et al., 2014). The efficacy of these balloons has not been established. Where COME is concerned, epidemiological studies tell us that host factors likely play a significant role. Yet, to date, human genetic-association studies have been underpowered, often poorly phenotyped and have an insufficient number of cohorts to enable the replication of their results.

In addition to human studies, animal models can be a powerful way to explore disease mechanisms. Mice have been used extensively to study chronic OM and its associated conditions, but none fully recapitulate the clinical features of COME or CSOM. The recent development of a mouse model of chronic otorrhoea, using surgical perforation of the tympanic membrane and Eustachian tube obstruction, represents a significant advance (Varsak and Santa Maria, 2016), but the model still lacks some important characteristics of human disease. The existence of large-scale mouse mutagenesis programs and new gene-editing techniques, such as CRISPR/Cas9, should also be harnessed to identify relevant mutants (Horii and Hatada, 2017), with a focus on identifying those models that faithfully recapitulate human disease and rejecting those that do not. To identify such models, we need to better understand how individuals are genetically predisposed to chronic OM and how the disease progresses. Any potentially informative model thus identified needs to then undergo detailed molecular and biological study to identify the underlying pathogenic mechanisms and the means by which these can be therapeutically manipulated to resolve effusion and/or otorrhoea.

We have little understanding of how current clinical treatments for chronic OM work at a molecular level. If we understood this better, perhaps we could offer more effective or reliable therapies. For example, antibiotics can sometimes stop otorrhoea in CSOM but we do not know to what extent they enable its long-term resolution and the healing of the tympanic membrane. We do not understand why the integrity of the tympanic membrane has such a profound effect on middle ear immunology. Creating a perforation of the tympanic membrane using grommets in children with COME leads to resolution of effusion but, conversely, surgical repair of a perforated tympanic membrane in individuals with CSOM leads to resolution. The use of bulla explants from animal models might provide an avenue for furthering our understanding of the role of the tympanic membrane in the aetiology of this disease.

Laboratory mice are widely used in OM research and, although most genetic models of chronic OM are syndromic, they provide important insights into, and a means by which to explore, the homeostatic mechanisms of the middle ear cleft. In particular, the innate immunity of the middle ear is likely to be relevant to the aetiology of non-syndromic chronic OM in humans, and we need therefore to better understand its mechanisms.

We should also not overlook the opportunities to study the transition between disease and health by extending the time course of induced AOM models. Our current chronic OM mouse models might pass the point where changes such as mucosal fibrosis are reversible. The prospects for generating a genetic mouse mutant that is an exact model of non-syndromic COME or CSOM are doubtful given species differences in size and anatomy, such as the presence of adenoidal lymphoid tissue and bulla mastoid cells in humans (Bhutta, 2012). Nevertheless, there is scope to discover more about the pathobiology of chronic OM in mutant mice, and to use them as novel translational models to validate candidate genes, pathways and experimental treatments. New techniques, such as designer-nuclease gene editors, make it possible to also engineer candidate OM genes in large animal species (Whitelaw et al., 2016), and the recent sequencing of the chinchilla genome (Shimoyama et al., 2016) opens up opportunities for further use of this species.

The challenge to overcome the research questions outlined here lies not only with clinicians and scientists, but perhaps also with research funding bodies, some of which may not have historically realised the prevalence and morbidity associated with chronic inflammation of the middle ear.

## Supplementary Material

Supplementary information
