# Supplementary material for: Understanding the aetiology and resolution of chronic otitis media from animal and human studies
Source: Dis Model Mech. 2017 Nov 1;10(11):1289–300. doi: 10.1242/dmm.029983 (PMC5719252; doi:10.1242/dmm.029983)
Supplement: Supplementary information [file dmm-10-029983-s1.pdf]

Supplementary Table S1

Current genetic mutant mouse models of chronic otitis media (OM). PCD = primary ciliary dyskinesia

mouse gene	reference	human syndrome or condition	adult mouse phenotypes additional to otitis media	craniofacial abnormality	Eustachian tube	bulla	bulla bacteria	inner ear	comment
**Down Syndrome**									
Ts65Dn Trisomy	(Han et al., 2009)	Down syndrome	behavioural and cognitive deficits, short narrow palate	short narrow palate		increase in goblet cells, effusion, fibrous proliferation	*Staph.* coagulase-negative, *Bordetella avium*, *Burkholderia cepacia*	labyrinth serous effusion; hair cells, stria vascularis & spiral ganglion normal	
Dp(16)1Yey Trisomy	(Bhutta et al., 2013)	Down syndrome				thickened mucoperiosteum, polyps			

Disease Models & Mechanisms • Supplementary information

**Primary Ciliary Dyskinesia (PCD)**									
Mdnah5 -/-	(Ibanez-Tallon et al., 2002)	PCD	hydrocephaly, growth retardation, mortality 2-3 weeks, situs inversus	domed head					
Dnahc11 iv/iv	(Lucas et al., 2012)	PCD	situs inversus, rhinitis, sinusitis						immotile tracheal cilia
Cby -/-	(Voronina et al., 2009)	PCD	rhinitis, growth retardation, post natal mortality by d30, anaemia, reduced subcutaneous fat, no situs inversus.						cilia deficiency and motility deficits, experimental challenge with *P. aeruginosa*
Odf2 ΔEx6,7/ΔEx6,7	(Kunimoto et al., 2012)	PCD	rhinitis, stertor, growth retardation, infertility, hydrocephaly variable						
Ttll1 -/-	(Vogel et al., 2012)	PCD not known with this mutation	PCD rhinosinusitis, male infertility, normal lungs,			suppurative exudate			no hydrocephalus, no situs inversus

Disease Models & Mechanisms • Supplementary information

Spag6 -/-	(Li et al., 2014)	PCD	hydrocephalus, sperm motility defects, morbidity and mortality after 6 months		normal acute angle to nasopharynx	no increase in goblet cell or mucin expression, loss of bulla cilia & disordered arrays; by 6 months cholesterol crystals, tympanosclerosis, mucosal capillary hyperplasia	PCR for human otopathogens *H. infle*, *S. pneumo*, negative *M. catar* positive by PCR & culture		
Ulk4 -/-	(Vogel et al., 2012)	PCD	PCD, rhinosinusitis, hydrocephalus, mortality < 4 months	hydrocephalus		suppurative OM			
Kif27 -/-	(Vogel et al., 2012)	PCD	PCD, rhinosinusitis, hydrocephalus, mortality < 8 weeks	hydrocephalus		suppurative OM			
Stk36 -/-	(Vogel et al., 2012)	PCD	PCD hydrocephalus, morbidity mortality < 6 weeks	hydrocephalus		suppurative OM without rhinosinusitis		inflammation extends into inner ear (and meninges)	
Dpcd -/-	(Vogel et al., 2012)	PCD	PCD, rhinosinusitis, hydrocephalus, male infertility	hydrocephalus		suppurative OM			

Disease Models & Mechanisms • Supplementary information

**Mucopolysaccharidosis**									
Ids KO	(Hong et al., 2012)	mucopolysaccharidosis type II, Hunter syndrome X-linked				TM thickened, microCT opacity, bulla exudate		minimal loss of hair cells, reduced ganglia	enzyme replacement therapy study
Naglu -/-	(Heldermon et al., 2007)	mucopolysaccharidosis type III, Sanfilippo syndrome	behavioural and sensory deficits, reduced life span			lysosomal storage in mucosa, osteocytes, chondrocytes & inflammatory cells		storage disease present in spiral ligament	
Idua -/-	(Schachern et al., 2007)	mucopolysaccharidosis type I				mucosa thickened and granulation tissue formation		storage disease present in spiral ligament	
Gus mps2J/mps2J	(Vogler et al., 2001)	mucopolysaccharidosis type VII	reduced survival, reduced lifespan, skeletal dysostosis, thickened tail	blunt nose		storage disease in mucosal connective tissue, osteocytes chondrocytes, thickened TM			

Disease Models & Mechanisms • Supplementary information

**22q11.2 Deletion Syndrome (DiGeorge Syndrome or Velo-Cardio-Facial Syndrome)**									
Df1/+	(Fuchs et al., 2013)	22q11.2 Deletion Syndrome	cardiovascular abnormalities, thymic or parathyroid defects	none reported	levator veli palatini hypoplasia	thickened mucosa, vascularization, reduced numbers of ciliated cells	*E. coli, Lactococcus lactis, Pantoea sp.*	no hair cell loss detected	
Tbx/+	Fuchs et al., 2015	22q11.2 Deletion Syndrome	cardiovascular abnormalities		levator veli palatini hypoplasia	mucosa proliferation, vascularization, effusion, increase in goblet cells			

**Hypohidrotic ectodermal dysplasia**									
Eda Ta/Ta, Eda Ta/Y, Edar dlJ/dlJ	(Azar et al., 2016)	Hypohidrotic Ectodermal dysplasia (HED) OMIM *EDA* 300451; *EDAR* 604095	rhinitis, nasopharyngitis, otitis media, HED, loss of nasal and nasopharyngeal submucosal glands		secondary dilation	mucosal thickening, polyps, foreign body granuloma	Gemella sp. Enterococcus sp. E. coli, Staphylococcus sp., Staph. aureus		
IKB αΔN	(Schmidt-Ullrich et al., 2001)		HED, immune defects lymph nodes absent, leukocytosis, impaired macrophage function, growth retardation	domed head		mucosal thickening	*Staphylococcus aureus*	normal	*Leishmania* challenge

Disease Models & Mechanisms • Supplementary information

**Other syndromes**									
Porcn +/del	(Biechele et al., 2013)	Focal Dermal Hypoplasia (Goltz Syndrome, OMIM#305600)	rhinitis, bronchopneumonia, mild hydrocephalus, dermal and skeletal defects			bulla exudate			cilia defect suspected
Chd7 Ome/+	(Tian et al., 2012)	CHARGE syndrome abnormalities of eye, heart, choana, genitalia, middle and inner ear	keratitis sicca, growth retardation, circling behaviour	skull height and nose length ratios higher	increased lateral angulation	increased goblet cells, reduced ciliated cells	Streptococci present in mutants and wild-type. PCR for human *H. influ*, *S. pneumo*, *M. catarrh* negative	normal hair cells and spiral ganglia	*Muc5AC*, *Muc5B*, *Tgfb1*
Lmna Dhe/+	(Zhang et al., 2012)	*LMNA* laminopathies includes Hutchinson-Gilford progeria syndrome, pinna & external ear canal anomaly, conductive hearing loss	epidermal dysplasia, craniofacial defects and reduced pinna, hyperphosphataemia and increased calcium x phosphate product in female mutants	fused basicranium	increased lateral angulation, increased width of bony segment more horizontal	mucosal thickening increased goblet cells, haemorrhage, erythrophagocytosis, cholesterol granuloma, abscessation			bulla *Tnfa*, *NF-kB*, *Tgfa* expression increased, functional assay peritoneal macrophages normal

Disease Models & Mechanisms • Supplementary information

Ets1 +/- and *Fli1* +/-	(Carpinelli et al., 2015)	Jacobsen syndrome	*Ets1* perinatal mortality, *Ets* and *Fli1* thrombocytopenia	short nasal bone, malformed septum		inflamed mucosa, small bullae, deformed stapes		none detected	
Mcph1 tm1a/tm1a	(Chen et al., 2013)	Microcephaly and mental retardation	homozygotes infertile, ocular abnormalities, increased B cells & micronucleated normochromic erythrocytes	small skull	normal	thickened mucoperiosteum	*Streptococcus sp.*		cilia defect suspected
Eya4 -/-	(Depreux et al., 2008)	sensoineural hearing loss, cardiomyopathy		none reported	reduced size, malposition	reduced bulla size, mucosal hyperplasia, reduced cilia, increased goblet cells			azithromycin treatment has no effect on phenotype
Phex Hyp-Duk/Y	(Han et al., 2012)	X-linked hypophosphatemic rickets	small body size and short tail	slightly abnormal skull shape	hyperplasia of ciliated epithelium	increased goblet cells, reduced ciliated cells, overlaid by patches mucin-like material	*Staph.* coagulase negative		

Disease Models & Mechanisms • Supplementary information

Anatomical defects									
Rpl38 Ts/+	(Noben-Trauth and Latoche, 2011)		skeletal abnormality, short misshapen tail, hyperphosphataemia	shortened nose	enlarged	polyps, cholesterol granuloma, mineralization, ectopic calcification, osteogenesis	*Staphylococcus* spp, *Enterococcus* sp.	no abnormality	rescued by Rpl38 cDNA
Sh3pxd2b nee homozygote	(Yang et al., 2011)		glaucoma, growth retardation, adipose deficits, hearing impairment	domed head, rounded skull, short maxilla and nose	inflammatory cells in ET lumen, increased lateral with concurrent reduced horizontal angulation	thickened fibrous mucosa with increased goblet cells and decreased ciliated cells, TM thickened		inner ear inflammation minor	increased *Tnf* and *Tlr2*

**TGF-β signalling pathway**									
Fbxo11 Jf/+	(Hardisty et al., 2003, Hardisty-Hughes et al., 2006)		homozygote cleft palate, eyelids open at birth, heterozygotes growth retardation	short snout	New-born and d50 mice ET narrowed and misshapen	bulla reduced size, mucosa dilated capillaries & lymphatics, polyps, calcification in mice > 11months		abnormal endocochlear potentials, possible impaired stria function	
Tgif -/-	(Tateossian et al., 2013)	holoprosencephaly	homozygote placental defect and minor hydrocephalu	minor skull shortening and reduced size	normal acute angle to nasopharynx	thickened mucoperiosteum, increased goblet cells		none at 2 months	bulla fluids elevated MMP, VEGF, TNFa

			s, growth retarded						
**Innate immunity**									
Mecom (*Evi1*) *Jbo*/+	(Azar et al., 2016, Bhutta et al., 2014, Cheeseman et al., 2011, Hood et al., 2016, Parkinson et al., 2006, Xu et al., 2012)		extra digit, derepression of NF-kB signalling			mucosa thickened polyps, vascularizatio n	*Staph* sp., *Staph aureus, Proteus mirabilis, E. coli, Moraxella* sp.		bulla fluids elevated cytokines and VEGF. experimental NTHi infection
Tlr4 Lps-d/Lps-d	MacArthur et al., 2006				lumen filled by exudate	mucosal fibrosis, granulation tissue, vascularizatio n and bulla bone remodelling		round window and organ of Corti inflammation	
BpifA1 (*Splunc-1*) -/-	(Bartlett et al., 2015)				normal	mucosal thickening and polyps, TM thickened, mucopurulent OM	special stains for bacteria and fungi negative		
Dbh -/-	(Maison et al., 2010)		absence of measurable epinephrine / norepinephrin e, altered metabolism,		loss adrenergic modulation auditory tube function	exudate or fibrous tissue in the vicinity of the round window, thickening		physiological changes	susceptibility to infection via compromised local and systemic

Disease Models & Mechanisms • Supplementary information

			thermoregulation, cardiovascular tone, maternal behaviour. Deficits in motor function, learning & memory			round window			immune cell function	
Isl1Drsh/+	(Hilton et al., 2011)					mucosa thickened by granulation tissue, mucosa and tympanic membrane vascularised, fusion of malleus and incus	*Proteus* sp.	no abnormality		

**Miscellaneous**										
Nf2 KO	(Giovannini et al., 2000)	neurofibromatosis	osteosarcoma, molar eruption delayed or absent, growth retardation, reduced survival			otitis media present but not described in detail				

Disease Models & Mechanisms • Supplementary information

Oxgr1 -/-	(Kerschner et al., 2013)					mucosal fibrosis, haemosiderin laden macrophages, mineralizatio n of ossicles	Gram stains for bacteria & PCR for human otopathogens *H. influe*, *S.pneumo*, *M. catarr* are negative		upregulation of *Muc5B*, *Muc 19*

References for table S1

Azar, A., Piccinelli, C., Brown, H., Headon, D. & Cheeseman, M. (2016). Ectodysplasin signalling deficiency in mouse models of hypohidrotic ectodermal dysplasia leads to middle ear and nasal pathology. *Hum Mol Genet*.

Bartlett, J. A., Meyerholz, D. K., Wohlford-Lenane, C. L., Naumann, P. W., Salzman, N. H. & Mccray, P. B., Jr. (2015). Increased susceptibility to otitis media in a Splunc1-deficient mouse model. *Dis Model Mech,* 8**,** 501-8.

Bhutta, M. F., Cheeseman, M. T. & Brown, S. D. (2014). Myringotomy in the Junbo mouse model of chronic otitis media alleviates inflammation and cellular hypoxia. *Laryngoscope,* 124**,** E377–E383.

Bhutta, M. F., Cheeseman, M. T., Herault, Y., Yu, Y. E. & Brown, S. D. (2013). Surveying the Down syndrome mouse model resource identifies critical regions responsible for chronic otitis media. *Mamm Genome,* 24**,** 439-45.

Biechele, S., Adissu, H. A., Cox, B. J. & Rossant, J. (2013). Zygotic Porcn paternal allele deletion in mice to model human focal dermal hypoplasia. *PLoS One,* 8**,** e79139.

Carpinelli, M. R., Kruse, E. A., Arhatari, B. D., Debrincat, M. A., Ogier, J. M., Bories, J. C., Kile, B. T. & Burt, R. A. (2015). Mice Haploinsufficient for Ets1 and Fli1 Display Middle Ear Abnormalities and Model Aspects of Jacobsen Syndrome. *Am J Pathol,* 185**,** 1867-76.

Cheeseman, M. T., Tyrer, H. E., Williams, D., Hough, T. A., Pathak, P., Romero, M. R., Hilton, H., Bali, S., Parker, A., Vizor, L., et al. (2011). HIF-VEGF Pathways Are Critical for Chronic Otitis Media in Junbo and Jeff Mouse Mutants. *PLoS Genet,* 7**,** e1002336.

Chen, J., Ingham, N., Clare, S., Raisen, C., Vancollie, V. E., Ismail, O., Mcintyre, R. E., Tsang, S. H., Mahajan, V. B., Dougan, G., et al. (2013). Mcph1-deficient mice reveal a role for MCPH1 in otitis media. *PLoS One,* 8**,** e58156.

Depreux, F. F., Darrow, K., Conner, D. A., Eavey, R. D., Liberman, M. C., Seidman, C. E. & Seidman, J. G. (2008). Eya4-deficient mice are a model for heritable otitis media. *J Clin Invest,* 118**,** 651-8.

Fuchs, J. C., Zinnamon, F. A., Taylor, R. R., Ivins, S., Scambler, P. J., Forge, A., Tucker, A. S. & Linden, J. F. (2013). Hearing loss in a mouse model of 22q11.2 Deletion Syndrome. *PLoS One,* 8**,** e80104.

Giovannini, M., Robanus-Maandag, E., Van Der Valk, M., Niwa-Kawakita, M., Abramowski, V., Goutebroze, L., Woodruff, J. M., Berns, A. & Thomas, G. (2000). Conditional biallelic Nf2 mutation in the mouse promotes manifestations of human neurofibromatosis type 2. *Genes Dev,* 14**,** 1617-30.

Han, F., Yu, H., Li, P., Zhang, J., Tian, C., Li, H. & Zheng, Q. Y. (2012). Mutation in Phex gene predisposes BALB/c-Phex(Hyp-Duk)/Y mice to otitis media. *PLoS One,* 7**,** e43010.

Han, F., Yu, H., Zhang, J., Tian, C., Schmidt, C., Nava, C., Davisson, M. T. & Zheng, Q. Y. (2009). Otitis media in a mouse model for Down syndrome. *Int J Exp Pathol,* 90**,** 480-8.

Hardisty, R. E., Erven, A., Logan, K., Morse, S., Guionaud, S., Sancho-Oliver, S., Hunter, A. J., Brown, S. D. & Steel, K. P. (2003). The deaf mouse mutant Jeff (Jf) is a single gene model of otitis media. *J Assoc Res Otolaryngol,* 4**,** 130-8.

Hardisty-Hughes, R. E., Tateossian, H., Morse, S. A., Romero, M. R., Middleton, A., Tymowska-Lalanne, Z., Hunter, A. J., Cheeseman, M. & Brown, S. D. (2006). A mutation in the F-box gene, Fbxo11, causes otitis media in the Jeff mouse. *Hum Mol Genet,* 15**,** 3273-9.

Heldermon, C. D., Hennig, A. K., Ohlemiller, K. K., Ogilvie, J. M., Herzog, E. D., Breidenbach, A., Vogler, C., Wozniak, D. F. & Sands, M. S. (2007). Development of sensory, motor and behavioral deficits in the murine model of Sanfilippo syndrome type B. *PLoS ONE,* 2**,** e772.

Hilton, J. M., Lewis, M. A., Grati, M., Ingham, N., Pearson, S., Laskowski, R. A., Adams, D. J. & Steel, K. P. (2011). Exome sequencing identifies a missense mutation in Isl1 associated with low penetrance otitis media in dearisch mice. *Genome Biol,* 12**,** R90.

Hong, S. H., Chu, H., Kim, K. R., Ko, M. H., Kwon, S. Y., Moon, I. J., Chung, W. H., Cho, Y. S., Kim, C. H., Suh, M. W., et al. (2012). Auditory characteristics and therapeutic effects of enzyme replacement in mouse model of the mucopolysaccharidosis (MPS) II. *Am J Med Genet A,* 158A**,** 2131-8.

Hood, D., Moxon, R., Purnell, T., Richter, C., Williams, D., Azar, A., Crompton, M., Wells, S., Fray, M., Brown, S. D., et al. (2016). A new model for non-typeable Haemophilus influenzae middle ear infection in the Junbo mutant mouse. *Dis Model Mech,* 9**,** 69-79.

Ibanez-Tallon, I., Gorokhova, S. & Heintz, N. (2002). Loss of function of axonemal dynein Mdnah5 causes primary ciliary dyskinesia and hydrocephalus. *Hum Mol Genet,* 11**,** 715-21.

Kerschner, J. E., Hong, W., Taylor, S. R., Kerschner, J. A., Khampang, P., Wrege, K. C. & North, P. E. (2013). A novel model of spontaneous otitis media with effusion (OME) in the Oxgr1 knock-out mouse. *Int J Pediatr Otorhinolaryngol,* 77**,** 79-84.

Kunimoto, K., Yamazaki, Y., Nishida, T., Shinohara, K., Ishikawa, H., Hasegawa, T., Okanoue, T., Hamada, H., Noda, T., Tamura, A., et al. (2012). Coordinated ciliary beating requires Odf2-mediated polarization of basal bodies via basal feet. *Cell,* 148**,** 189-200.

Li, X., Xu, L., Li, J., Li, B., Bai, X., Strauss, J. F., 3rd, Zhang, Z. & Wang, H. (2014). Otitis media in sperm-associated antigen 6 (Spag6)-deficient mice. *PLoS One,* 9**,** e112879.

Liao, J., Kochilas, L., Nowotschin, S., Arnold, J. S., Aggarwal, V. S., Epstein, J. A., Brown, M. C., Adams, J. & Morrow, B. E. (2004). Full spectrum of malformations in velo-cardio-facial syndrome/DiGeorge syndrome mouse models by altering Tbx1 dosage. *Hum Mol Genet,* 13**,** 1577-85.

Lucas, J. S., Adam, E. C., Goggin, P. M., Jackson, C. L., Powles-Glover, N., Patel, S. H., Humphreys, J., Fray, M. D., Falconnet, E., Blouin, J. L., et al. (2012). Static respiratory cilia associated with mutations in Dnahc11/DNAH11: a mouse model of PCD. *Hum Mutat,* 33**,** 495-503.

Macarthur, C. J., Hefeneider, S. H., Kempton, J. B. & Trune, D. R. (2006). C3H/HeJ mouse model for spontaneous chronic otitis media. *Laryngoscope,* 116**,** 1071-9.

Maison, S. F., Le, M., Larsen, E., Lee, S. K., Rosowski, J. J., Thomas, S. A. & Liberman, M. C. (2010). Mice lacking adrenergic signaling have normal cochlear responses and normal resistance to acoustic injury but enhanced susceptibility to middle-ear infection. *J Assoc Res Otolaryngol,* 11**,** 449-61.

Noben-Trauth, K. & Latoche, J. R. (2011). Ectopic mineralization in the middle ear and chronic otitis media with effusion caused by RPL38 deficiency in the Tail-short (Ts) mouse. *J Biol Chem,* 286**,** 3079-93.

Parkinson, N., Hardisty-Hughes, R. E., Tateossian, H., Tsai, H. T., Brooker, D., Morse, S., Lalane, Z., Mackenzie, F., Fray, M., Glenister, P., et al. (2006). Mutation at the Evi1 locus in Junbo mice causes susceptibility to otitis media. *PLoS Genet,* 2**,** e149.

Schachern, P. A., Cureoglu, S., Tsuprun, V., Paparella, M. M. & Whitley, C. B. (2007). Age-related functional and histopathological changes of the ear in the MPS I mouse. *Int J Pediatr Otorhinolaryngol,* 71**,** 197-203.

Schmidt-Ullrich, R., Aebischer, T., Hulsken, J., Birchmeier, W., Klemm, U. & Scheidereit, C. (2001). Requirement of NF-kappaB/Rel for the development of hair follicles and other epidermal appendices. *Development,* 128**,** 3843-53.

Tateossian, H., Morse, S., Parker, A., Mburu, P., Warr, N., Acevedo-Arozena, A., Cheeseman, M., Wells, S. & Brown, S. D. (2013). Otitis media in the Tgif knockout mouse implicates TGFbeta signalling in chronic middle ear inflammatory disease. *Hum Mol Genet,* 22**,** 2553-65.

Tian, C., Yu, H., Yang, B., Han, F., Zheng, Y., Bartels, C. F., Schelling, D., Arnold, J. E., Scacheri, P. C. & Zheng, Q. Y. (2012). Otitis Media in a New Mouse Model for CHARGE Syndrome with a Deletion in the Chd7 Gene. *PLoS ONE,* 7**,** e34944.

Disease Models & Mechanisms • Supplementary information

Vogel, P., Read, R. W., Hansen, G. M., Payne, B. J., Small, D., Sands, A. T. & Zambrowicz, B. P. (2012). Congenital hydrocephalus in genetically engineered mice. *Vet Pathol,* 49**,** 166-81.
Vogler, C., Levy, B., Galvin, N., Sands, M. S., Birkenmeier, E. H., Sly, W. S. & Barker, J. (2001). A novel model of murine mucopolysaccharidosis type VII due to an intracisternal a particle element transposition into the beta-glucuronidase gene: clinical and pathologic findings. *Pediatr Res,* 49**,** 342-8.
Voronina, V. A., Takemaru, K., Treuting, P., Love, D., Grubb, B. R., Hajjar, A. M., Adams, A., Li, F. Q. & Moon, R. T. (2009). Inactivation of Chibby affects function of motile airway cilia. *J Cell Biol,* 185**,** 225-33.
Warren, M., Wang, W., Spiden, S., Chen-Murchie, D., Tannahill, D., Steel, K. P. & Bradley, A. (2007). A Sall4 mutant mouse model useful for studying the role of Sall4 in early embryonic development and organogenesis. *Genesis,* 45**,** 51-8.
Xu, X., Woo, C. H., Steere, R. R., Lee, B. C., Huang, Y., Wu, J., Pang, J., Lim, J. H., Xu, H., Zhang, W., et al. (2012). EVI1 Acts as an Inducible Negative-Feedback Regulator of NF-kappaB by Inhibiting p65 Acetylation. *J Immunol,* 188**,** 6371-80.
Yang, A., Walker, N., Bronson, R., Kaghad, M., Oosterwegel, M., Bonnin, J., Vagner, C., Bonnet, H., Dikkes, P., Sharpe, A., et al. (2000). p73-deficient mice have neurological, pheromonal and inflammatory defects but lack spontaneous tumours. *Nature,* 404**,** 99-103.
Yang, B., Tian, C., Zhang, Z. G., Han, F. C., Azem, R., Yu, H., Zheng, Y., Jin, G., Arnold, J. E. & Zheng, Q. Y. (2011). Sh3pxd2b mice are a model for craniofacial dysmorphology and otitis media. *PLoS ONE,* 6**,** e22622.
Zhang, Y., Yu, H., Xu, M., Han, F., Tian, C., Kim, S., Fredman, E., Zhang, J., Benedict-Alderfer, C. & Zheng, Q. Y. (2012). Pathological Features in the Lmna(Dhe/+) Mutant Mouse Provide a Novel Model of Human Otitis Media and Laminopathies. *Am J Pathol*.
